# Anti-inflammatory and antioxidant potential, *in vivo* toxicity, and polyphenolic composition of *Eugenia selloi* B.D.Jacks. (pitangatuba), a Brazilian native fruit

**DOI:** 10.1371/journal.pone.0234157

**Published:** 2020-06-09

**Authors:** Josy Goldoni Lazarini, Marcelo Franchin, Jackeline Cintra Soares, Bruno Dias Nani, Adna Prado Massarioli, Severino Matias de Alencar, Pedro Luiz Rosalen

**Affiliations:** 1 Department of Physiological Sciences, Piracicaba Dental School, University of Campinas, Piracicaba, Sao Paulo, Brazil; 2 Faculty of Dentistry, Federal University of Alfenas - UNIFAL, Alfenas, Minas Gerais, Brazil; 3 Department of Agri-Food Industry, Food and Nutrition, “Luiz de Queiroz” College of Agriculture, University of São Paulo, Piracicaba, Sao Paulo, Brazil; 4 Biological Sciences Graduate Program, Federal University of Alfenas - UNIFAL, Alfenas, Minas Gerais, Brazil; Institute for Biological Research "S. Stanković", University of Belgrade, SERBIA

## Abstract

Brazilian native fruits are a rich source of polyphenolic compounds that can act as anti-inflammatory and antioxidant agents. Here, we determined the polyphenolic composition, anti-inflammatory mechanism of action, antioxidant activity and systemic toxicity in *Galleria mellonella* larvae of *Eugenia selloi* B.D.Jacks. (synonym *Eugenia neonitida* Sobral) extract (*Es*e) and its polyphenol-rich fraction (F3) obtained through bioassay-guided fractionation. Phenolic compounds present in *Es*e and F3 were identified by LC-ESI-QTOF-MS. The anti-inflammatory activity of *Es*e and F3 was tested *in vitro* and *in vivo* through NF-κB activation, cytokine release and neutrophil migration assays. The samples were tested for their effects against reactive species (ROO•, O2•-, HOCl and NO•) and for their toxicity in *Galleria mellonella* larvae model. The presence of hydroxybenzoic acid, ellagitannins and flavonoids was identified. *Es*e and F3 reduced NF-κB activation, cytokine release and neutrophil migration, with F3 being three-fold more potent. Overall, F3 exhibited strong antioxidant effects against biologically relevant radicals, and neither *Es*e nor F3 were toxic to *G*. *mellonella* larvae. In conclusion, *Es*e and F3 revealed the presence of phenolic compounds that decreased the inflammatory parameters evaluated and inhibited reactive oxygen/nitrogen species. *E*. *selloi* is a novel source of bioactive compounds that may provide benefits for human health.

## Introduction

The Brazilian Atlantic rainforest is a rich ecosystem which has been extensively threatened by deforestation. With only 12% of the original area left, it shelters numerous fauna and flora species, including approximately 50% endemic edible fruit trees. Occurring in the Atlantic rainforest, Brazilian native fruit species are part of a yet unknown, neglected and underutilized biodiversity, especially for bioprospecting novel molecules with biological properties [1,2].

The genus *Eugenia* (Myrtaceae family) has approximately 400 native species occurring in the Atlantic rainforest. Brazilian native species have great economical potential for fresh fruit consumption; production of juice, jam and ice cream; as well as for pharmaceutical and nutritional use [2]. Recent studies reported that *Eugenia* spp. have anti-cancer (*E*. *uniflora* and *E*. *brasiliensis*), anti-inflammatory and antioxidant effects (*E*. *leitonii*, *E*. *brasiliensis*, *E*. *stipitata*, *E*. *myrcianthes* and *E*. *involucrata*), among others [2–5]. Currently, some traditional fruits and Brazilian native fruit species, such as *E*. *uniflora* and *Euterpe oleracea*, have been termed “superfruits” due to their high levels of phytonutrients (bioactive molecules) that can improve regular consumers’ health [6].

*Eugenia* fruit species contain a rich nutritional value (minerals and vitamins) and are a promising source of phytonutrients, such as carotenoids, flavanols (catechin, epicatechin), anthocyanidins (cyaniding, delphinidin, malvidin), flavonols (kaempferol, quercetin), phenolic acids (gallic acid), and others [2–6]. Scientific evidence suggests that regular intake of bioactive molecules contained in native fruits has been associated with a reduced risk of developing oxidative stress-related diseases, such as diabetes, obesity, cardiovascular disorders, cancer, arthritis, chronic inflammatory diseases etc [2–9]. The latter commonly go unnoticed and are triggered by oxidative stress generated by reactive oxygen / nitrogen species (ROS / RNS), resulting in cell damage and tissue injury [9]. ROS and RNS can overly activate an important signaling pathway associated with inflammation, the nuclear factor κB (NF-κB), and thereby they render the inflammatory process more destructive than resolutive [4,9,10]. Thus, the search for, and application of, novel bioactive compounds able to control the inflammatory process while modulating ROS/RNS generation is much needed.

Recently, our research group reported the anti-inflammatory and antioxidant activity of five Brazilian native fruit species [4]. *Eugenia selloi* B.D.Jacks. (synonym *Eugenia neonitida* Sobral), showed the strongest *in vivo* anti-inflammatory activity in mice model and thus was selected for further analysis in the present study. *E*. *selloi*, popularly known as “pitangatuba”, “pitangola”, “pitangão” or “pitanga-amarela”, is a shrub tree approximately two meters high, with dark green leaves. Its fruit is 4 cm long and 3 cm wide, has pleasant bittersweet taste with juicy pulp to consume *in natura* or in beverages, juices and jams [11]. While *E*. *selloi* has been used in folk medicine to treat skin depigmentation and vitiligo–because it contains a photosensitizing substance [11], its anti-inflammatory activity, ROS/RNS scavenging activity, and phytochemical composition, remain to be determined.

Thus, our study hypothesis was that the phytochemical compounds present in *E*. *selloi* have ROS/RNS scavenging effects and, consequently, inhibit important pro-inflammatory biomarkers. To test our hypothesis, we determined the phytochemical composition of *E*. *selloi* extract (*Es*e) and its polyphenol-rich fraction (F3) and tested them for their anti-inflammatory mechanism of action, antioxidant activity and systemic toxicity *in vivo*.

## Material and methods

### Reagents

The reagents were purchased from Supelco (Bellefonte, PA, USA): LC-18 SPE cartridges 2g. Tedia (Fairfield, OH, USA): formic acid. R&D Systems, Inc: TNF-α and CXCL2/MIP-2 kits. Applied Biological Materials Inc. (Richmond, BC,Canada): RAW 264.7 macrophages transfected with the *NF-κB-pLUC* gene. J.T. Baker (Phillipsburg, NJ, USA): acetonitrile, methanol and ethanol. Millipore Milli-Q System (Millipore SAS, Molsheim, France): purified water. Sigma-Aldrich (St. Louis, MO, USA): RPMI (Roswell Park Memorial Institute) 1640 medium, lipopolysaccharide (LPS) from *Escherichia coli* 0111:B4, DMSO (dimethylsulfoxide), carrageenan, dexamethasone, diaminofluorescein-2 (DAF-2), 3-(4,5-dimethylthiazol-2-yl)-2,5-diphenyltetrazolium bromide (MTT), (±)-6-hydroxy-2,5,7,8-tetramethylchroman-2-carboxylic acid (Trolox), sodium nitroprusside, sodium hypochlorite solution (NaOCl), nitrotetrazolium blue chloride (NBT), dibasic potassium phosphate, 2,2-azobis(2-methylpropionamidine) β-nicotinamide adenine dinucleotide (NADH), phenazine methosulfate (PMS), rhodamine 123, dihydrochloride (AAPH) and fluorescein sodium salt. Gibco (Grand Island, NY, USA): fetal bovine serum. MERCK KGaA (Frankfurt, Germany): Silicagel 60 (0,063–0,200 mm). Promega Corporation (Madison, WI, USA): luciferin. Amresco, Inc. (West Chester, Pensilvania, EUA): Lysis buffer TNT, mixture of TRIS BASE and Tween 20.

### Plant material and extract preparation

Access to *E*. *selloi* fruit was previously granted by the Council for Genetic Heritage Management (Brazilian Ministry of Environment; CGEN #AD4B64F). *E*. *selloi* B.D.Jacks. samples were collected several times in a farm named “Rare Fruits” (“*Frutas Raras*” in Portuguese), located in the city of Campina do Monte Alegre, São Paulo, Southeastern Brazil. The farm is in the Atlantic rainforest subtropical region (Cfb—Köppen climate classification), with an altitude of 612 meters (S 23° 53’ 57.06"; W 48° 51’ 24.68"). Fruit samples were collected from November 2015 to February 2016, and the specimen was identified by experts from the herbarium of the “Luiz de Queiroz” College of Agriculture, University of São Paulo (ESALQ/USP), Piracicaba, São Paulo, under voucher HPL 5279. Only intact (undamaged) fruit samples were collected, which were transported under refrigeration and subsequently washed with water. Fruit pulps were separated, frozen, lyophilized, and stored at -18°C until further analysis. For extract preparation, lyophilized *E*. *selloi* pulp (50 g) was mixed with ethanol and water (80:20, v/v; respectively). The mixture was submitted to three ultrasound cycles (30 min each), filtered to separate the liquid content, evaporated and lyophilized. *E*. *selloi* extract (*Ese*) was stored at -20°C until further use.

### *E*. *selloi* extract fractionation

*Es*e was submitted to a fractionation system by means of an open dry column chromatography using normal phase silica gel. To recover fractions with different polarities, the mixture of ethyl acetate:methanol:water (77:13:10, v/v) was applied onto the top of the column. After the solvent passed through the whole column, six fractions were recovered, evaporated and lyophilized. The fractions were monitored using Thin Layer Chromatography (TLC). Fluorescent compounds were visualized under ultraviolet (UV) light at 366 nm wavelength. *Es*e and its most active fraction (F3) were submitted to LC-ESI-QTOF-MS analysis.

### Phytochemical analysis

#### Sugar removal

To remove the sugar content of *Es*e, 500 mg of the extract was diluted with 5 mL of water and centrifuged (5000 x g for 15 min). The precipitate was dissolved in 5 mL of HCl solution (pH = 2.0) and filtered. In parallel, LC-18 SPE cartridges were conditioned with acidic water and methanol (pH = 2.0). Then, 5 mL of *Es*e diluted in HCl solution were added to each cartridge until the total liquid content passed through the column. The wash process was repeated with acidic water to remove the sugar content. Finally, the compounds of interest were eluted with methanol, and sugar-free *Es*e was recovered and stored at -20°C until further use.

#### High-resolution mass spectrometry analysis (LC-ESI-QTOF-MS)

LC-ESI-QTOF-MS analysis was carried out using a chromatograph (Shimadzu Co., Tokyo) with a quaternary pump LC-30AD, photodiode array detector (PDA) SPD-20A. Reversed phase chromatography was performed using Phenomenex Luna C18 column (4.6 x 250 mm x 5 μm). High-resolution mass spectrometry MAXIS 3G –Bruker Daltonics (Bruker Daltonics, Bremen, Germany) was fitted with a Z-electrospray (ESI) interface operating in negative ion mode with a nominal resolution of 60,000 m/z. Twenty microliters of *Es*e and F3 were injected into a liquid chromatography system. The conditions for the analysis of *Es*e were as follows: nebulizer at 2 Bar; dry gas at 8 L/min; temperature at 200 °C and HV at 4500 V. The mobile phase consisted of two solvents: (A) water/formic acid (99.75/0.25, v/v) and (B) acetonitrile/formic acid/water (80/0.25/19.75, v/v). The flow rate was 1 mL/min, and the gradient was initiated with 10% B, increasing to 20% B (10 min), 30% B (20 min), 50% B (30 min), 90% B (38 min), and decreasing to 10% B (45 min), completing after 55 min. The conditions for the analysis of F3 was similar to those of *Es*e, except for the mobile phase, which consisted of two solvents: water/formic acid (99.75/0.25, v/v) (A) and acetonitrile (100) (B). The flow rate was 1 mL/min, and the gradient was initiated with 10% solvent B, increasing to 30% B (20 min), 50% B (32 min), 95% B (38 min), 95% B (60 min), and decreasing to 10% B (75 min), completing after 80 min. External calibration was carried out using the software MAXIS 3G –Bruker Daltonics 4.3 to check for mass precision and data analysis. The identification of compounds was performed tentatively by comparison of exact mass, MS/MS mass spectra and molecular formulae available from the scientific literature.

### Evaluation of anti-inflammatory activity

#### Cell culture and viability *in vitro* assay

RAW 264.7 macrophages (ATCC^®^ TIB-71™) transfected with the *NF-κB-pLUC* gene to express luciferase were cultured in endotoxin-free RPMI 1640 medium supplemented with fetal bovine serum (10%, v/v), penicillin (100 U/mL), streptomycin sulfate (100 μg/mL) and L-glutamine (37 °C, 5% CO_2_). Macrophages were cultured in 96-well plates (5 × 10^5^ cells/mL) and incubated overnight. *Es*e (10, 30, 100, 300 and 1000 μg /mL) and F3 (0.1, 10, 30, 100 and 300 μg /mL), or culture media (negative control), were added to each well and incubated for 24 h. All groups were stimulated with 10 ng/mL lipopolysaccharide (LPS), except the negative control. After this period, the supernatant was removed and an MTT solution (0.3 mg/mL) was added to the wells. The plates remained incubated for 3 h (37 °C, 5% CO_2_). The supernatant was removed and DMSO was added. Absorbance was measured at 570 nm using an ELISA microplate reader.

#### NF-κB activation, and TNF-α and CXCL2/MIP-2 levels

Macrophage cells transfected were cultured in 24-well plates (3 × 10^5^ cells/mL). The cells were treated with *Es*e or F3 at 30, 100 and 300 μg/mL for 30 min before LPS stimulation (10 ng/mL) for 4 h, except the culture media control (negative control). After 4 h, cell lysis buffer and 25 μL of luciferase reagent (luciferin at 0.5 mg/mL) were added to each well. Luminescence was measured using a white microplate reader (SpectraMax M3, Molecular Devices). In addition, TNF-α and CXCL2/MIP-2 levels were determined according to the protocols provided by the manufacturers on an ELISA microplate reader. The results were expressed as pg/mL.

### *In vivo* anti-inflammatory assays

#### Animals

*In vivo* experiments were performed with male SPF (specific-pathogen free) C57BL/6JUnib mice, purchased from CEMIB / UNICAMP (Multidisciplinary Center for Biological Research, SP, Brazil), weighing between 22 and 25 g. All animals were housed *in vivarium* under humidity (40–60%) and temperature (22 ± 2 °C) control in 12 h light-dark cycle, with access to food and water *ad libitum*. For the experiment, all animals were deprived of food for 8 h before oral administration. This study complied with the National Council for Animal Experimentation Control guidelines for the care and use of animals in scientific experimentation (https://www.mctic.gov.br/mctic/opencms/institucional/concea/paginas/legislacao.html), according to the Brazilian Law 11,794, of October 8, 2008. All animals were euthanized by deepening anesthesia with Ketamine and Xylazine (300 mg/kg and 30 mg/kg, respectively) and, after, submitted to cervical dislocation. The study protocol was previously approved by the Institutional Ethics Committee on Animal Research at the University of Campinas (CEUA/UNICAMP, Protocol Number 4371–1, approved on 09/23/2016).

#### Inhibition of neutrophil migration

Mice received orally (via gavage) single doses of *Es*e (3 or 10 mg/kg) or F3 (3 or 10 mg/kg). The negative control group received oral administration of 0.9% saline (vehicle) and 2 mg/kg dexamethasone. After 1 h, all animals received an inflammatory challenge by intraperitoneal (i.p.) injection of the flogistic agent carrageenan (500 μg/cavity) for 4 h, except the vehicle group. Next, the animals were sacrificed, their peritoneal cavities were washed and recovered to count for the total number of leukocytes and neutrophils. The results were expressed as number of neutrophils per cavity. TNF-α and CXCL2/MIP-2 levels were determined using an ELISA microplate reader. The results were expressed as pg/mL.

### Evaluation of reactive oxygen/nitrogen species scavenging capacity (antioxidant activity)

#### Peroxyl radical (ROO^•^)

Briefly, 30 μL of *Es*e or F3 plus 60 μL of fluorescein and 110 μL of an AAPH solution were transferred to a microplate. The reaction was performed at 37 °C and absorbance was measured every minute for 2 h at 485 nm (excitation) and 528 nm (emission) on a microplate reader (Molecular Devices, LLC, Sunnyvale, CA, USA). Trolox standard was used at concentrations ranging from 12.5 to 400 μM. The results were expressed as μmol/Trolox equivalents per g of extract/fraction (*Es*e and F3) [4].

#### Superoxide anion (O_2_^•-^)

The capacity of *Es*e and F3 to scavenge O_2_^•-^ generated by the NADH/PMS system was determined as previously described [12]. Briefly, 100 μL of NADH, 50 μL of NBT, 100 μL of *Es*e or F3 (at different concentrations) and 50 μL of PMS were mixed in the microplate. The assay was performed at 25 °C and absorbance was measured after 5 min at 560 nm. A control was prepared replacing the sample with the buffer, and a blank was prepared for each sample dilution replacing PMS and NADH with the buffer. Absorbance was measured in a microplate reader (Molecular Devices, LLC, Sunnyvale, CA, USA) and the results were expressed as IC_50_, the mean concentration (μg/mL) of *Es*e or F3 required to quench 50% of the superoxide radicals [12].

#### Hypochlorous acid (HOCl)

The HOCl scavenging activity of *Es*e and F3 was measured by monitoring their effects on HOCl-induced oxidation of dihydrorhodamine 123 (DHR) to rhodamine 123, with modifications. HOCl was prepared using a 1% NaOCl solution, adjusting the pH to 6.2. The reaction mixture contained *Es*e or F3 at different concentrations, phosphate buffer (pH 7.4), DHR, and HOCl, in a final volume of 300 μL. The assay was carried out at 37 °C in a microplate reader (Molecular Devices, LLC, Sunnyvale, CA, USA) and fluorescence was measured immediately at 528 ± 20 nm (emission) and 485 ± 20 nm (excitation). The results were expressed as IC_50_ (μg/mL) of *Es*e or F3 [4].

#### Nitric oxide (NO^•^)

The nitric oxide (NO^•^) activity of *Ese* and F3 was determined using diaminofluorescein-2 (DAF-2) as a NO^•^ probe. Briefly, 50 μL of *Es*e or F3 plus 50 μL of SNP solution, 50 μL of buffer and 50 μL of DAF solution were added to the wells (96-well plate). Changes in fluorescence (excitation = 495 nm, emission = 515 nm) were measured in a microplate reader (Molecular Devices, LLC, Sunnyvale, CA, USA) over a 120-min period at 5-min intervals. The results were expressed as IC_50_ (μg/mL) of *Es*e or F3 [4].

### Systemic toxicity

#### *Galleria mellonella* model

To provide preliminary evidence on the potential toxic effects of *Es*e and F3, their acute systemic toxicity was determined in a *G*. *mellonella* L. model. *G*. *mellonella* larvae were kindly provided by L. G. Leite from the Biological Institute, Department of Agriculture and Supply (Campinas, São Paulo, Brazil). The larvae were maintained at 37°C in a BOD incubator until use. Six equal increasing doses of *Es*e and F3 (0.01, 0.1, 0.3, 1, 3 and 10 g/kg) were tested. Larvae with no signs of melanization, weighing 200 to 300 mg, were randomly selected for each group (n = 15). An aliquot of 10 μL of *Es*e, F3 or control (0.9% NaCl, w/v) were injected into their hemocoel via the last left proleg using a Hamilton syringe (Hamilton, Reno, NV). The larvae were incubated at 30 °C and their survival was monitored at selected intervals for up to 72 h. Larvae with no movements upon touch were counted as dead [13].

### Statistical analysis

The data were checked for normality and submitted to one-way analysis of variance (ANOVA) followed by Tukey’s post-hoc test. The results were expressed as mean ± standard deviation (SD). In *in vivo* toxicity assays, survival curves of treated and untreated *G*. *mellonela* larvae were compared using the Log-rank (Mantel-Cox) test. The results were considered significant at *P* ≤ 0.05.

## Results

### Phytochemical analysis

The phenolic compounds were tentatively identified by analysis of their exact masses, MS/MS spectra and molecular formulas using High-Resolution Mass Spectrometry analysis (LC-ESI-QTOF-MS). As seen in Table 1, the chemical analysis revealed 16 compounds in *Es*e, 13 compounds in F3, and 8 compounds present in both samples. Overall, hydroxybenzoic acid, flavanols, ellagitannins, flavone and flavonols were detected in the samples. The chemical fractionation process is shown in supporting information S1 Fig. Visual aspect of *Es*e, chemical fractionation, yield and thin layer chromatography.

**Table 1:** Chemical analysis of *Es*e and F3 by High-resolution mass spectrometry (LC-ESI-QTOF-MS).

Compound	Rt (min)	Molecular formula	[M-H]^-^	MS fragments (m/z)	*Es*e	F3
**Hydroxybenzoic acid**						
Gallic acid	4.6	C_7_H_6_O_5_	169.0105	**125.0209**, 117.9917, 140.5531, 150.3022	**-**	**+**
Syringic acid hexoside	14.7	C_15_H_20_O_10_	359.1259	153.0888, **197.0771**, 212,6754, 119.0322	**+**	**+**
Sinapic acid-*O*-hexoside I	19.9	C_17_H_22_O_10_	385.1409	**179.1035**, 119.0319, 135,1143, 223.0879	**+**	**+**
Sinapic acid-*O*-hexoside II	20.1	C_17_H_22_O_10_	385.1409	**179.1044**, 135.1152, 113.0207, 223.0874	**-**	**+**
**Flavanols**						
(Epi)catechin	11.4	C_15_H_14_O_6_	289.0726	**289.0726**, 245.0814, 179.0340, 205.0473	**+**	**-**
(Epi)catechin derivative	12.8	C_15_H_14_O_6_	401.0886	**289.0725**, 245.0825, 205.0508, 179.0342	**+**	**-**
**Ellagitannins**						
Ellagic acid	20.2	C_14_C_6_O_8_	300.9990	**300.9999**, 229.5014, 255.2311, 257.0124	**+**	**-**
Galloyl-HHDP-hexoside	8.3	C_27_H_22_O_18_	633.0559	300.9922, **301.9946**, 169.0092, 463.0512, 481.0551	**-**	**+**
Di-HHDP-galloyl-glucose derivate I	10.9	C_41_H_27_O_26_	467.0369 [M − 2H]^2-^	**275.0200**, 343.0106, 300.9988,169.0503	**+**	**+**
Di-HHDP-galloyl-glucose I	10.9	C_41_H_27_O_26_	935.0797	**299.0203**, 275.0199, 300.9994, 633.0743	**+**	**-**
Di-HHDP-galloyl-glucose derivate II	11.8	C_41_H_27_O_26_	467.0372 [M − 2H]^2-^	**275.0200**, 169.0146, 301.000, 343.0100	**+**	**-**
Di-HHDP-galloyl-glucose II	11.8	C_41_H_27_O_26_	935.0806	**275.0200**, 299.0206, 633.0737, 300.99898	**+**	**-**
Di-HHDP-galloyl-glucose derivate III	16.6	C_41_H_27_O_26_	467.0365 [M − 2H]^2-^	**300.9996**, 423.1872, 169.0141, 275.0247	**+**	**-**
**Flavone**						
Apigenin-7-*O*-glucoside	14.0	C_21_H_20_O_11_	431.1810	205.1177, 121.0290, 119.0320, **269.2089,** 153.0176, 241.9622, 311.4209	**-**	**+**
**Flavonols**						
Quercetin-*O*-hexoside I	20.2	C_21_H_20_O_12_	463.0887	**301.0328**,271.02445,463.0884, 243.0800	**+**	**+**
Quercetin-*O*-hexoside II	20.5	C_21_H_20_O_12_	463.0890	**301.0352**, 271.0248, 243.0802, 463.0889	**+**	**+**
Quercetin-*O*-(*O*-galloyl)-hexoside	21.2	C_28_H_24_O_16_	615.0996	**301.0357**, 271.0248, 179.0982, 151.0039	**+**	**+**
Quercetin-*O*-acethylhexoside	22.4	C_23_H_22_O_13_	505.0994	221.0238, **301.0335**, 506.1011, 463.0024	**+**	**-**
Quercetin-3-malonylglucoside	22.4	C_24_H_22_O_15_	549.0889	300.0279, **301.0328**, 271.0249, 243.0807	**+**	**+**
Kaempferol-3-*O*-glucoside	23.2	C_21_H_20_O_11_	447.0940	**285.0335**, 284.0335, 255.0299, 227.0851	**+**	**+**

Bold values indicate the main fragments; Rt = retention time; [M-H]- (negative ionization mode) experimental mass of compound; + detected/—not detected.

### *In vitro* anti-inflammatory assays

#### Viability assay, NF-κB activation, TNF-α and CXCL2/MIP-2 levels

**Fig 1:**
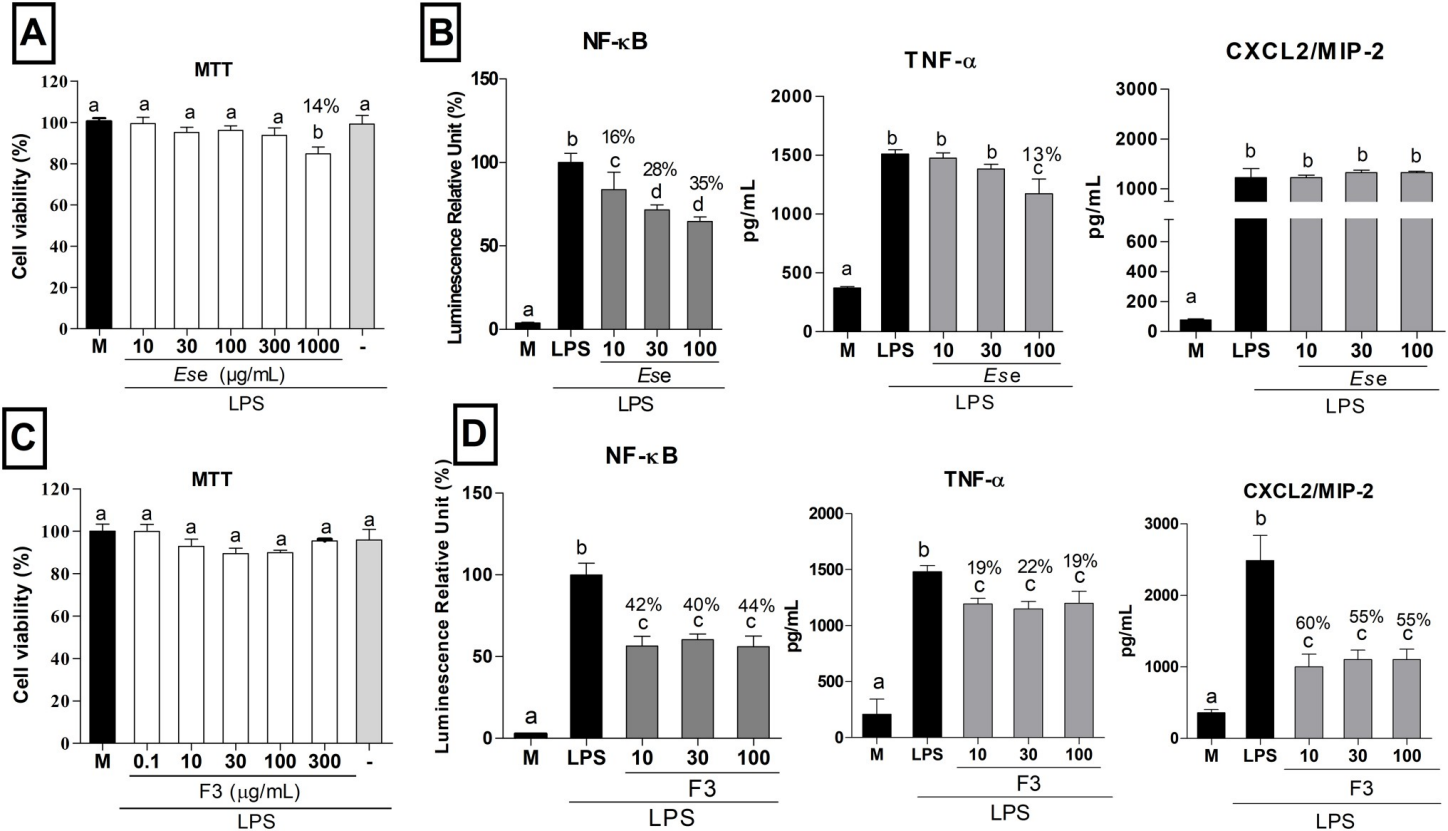
Effects of *Es*e and F3 on cell viability and inflammation markers in macrophages. (A and C) Evaluation of RAW 264.7 macrophages treated with culture medium (M), LPS (10 ng/mL,-), *Es*e (10, 30, 100, 300 and 1000 μg/mL) and F3 (0.1, 10, 30, 100 and 300 μg/mL) for 24h. (B and D) Evaluation of NF-κB activation and TNF-α and CXCL2/MIP-2 release in RAW 264.7 macrophages. The results were expressed as mean ± SD, *n* = 4. Different letters indicate statistical difference and the symbol % indicates a decrease in NF- kB activation. One-way ANOVA followed by Tukey’s post-hoc test, *P* < 0.05.

As seen in Fig 1A, treatment with *Es*e at concentrations up to 300 μg/mL did not significantly affect cell viability in LPS-stimulated macrophages as compared to the culture media control (*P* > 0.05). However, cells treated with *Es*e at 1000 μg/mL showed reduced cell viability (14%) as compared to the control (*P* < 0.05). As expected, LPS treatment (10 ng/mL) did not affect cell viability (*P* > 0.05).

Fig 1B shows that treatment with *Es*e (10, 30 and 100 μg/mL) significantly reduced NF-κB activation by 16%, 28% and 35%, respectively, as compared to LPS-treated cells. Moreover, treatment with *Ese* reduced TNF-α levels by 13% at 100 μg/mL (*P* < 0.05), but no significant differences were observed at the concentrations of 10 and 30 μg/mL (*P* > 0.05). Likewise, treatment with *Ese* did not affect the release of CXCL2/MIP-2 by macrophages at any tested concentration as compared to LPS-treated cells (*P* > 0.05, Fig 1B). The polyphenol-rich fraction (F3) from *Es*e did not affect macrophage viability at any tested concentration as compared to the culture media control and LPS-treated cells (*P* > 0.05; Fig 1C). Macrophage cells treated with F3 at 10, 30 and 100 μg/mL had NF-κB activation significantly reduced (42%, 40% and 44%, respectively) as compared to LPS-treated cells (*P* < 0.05; Fig 1D). At 10, 30 and 100 μg/mL, F3 reduced (19%, 22% and 19%, respectively) the release of TNF-α compared to the control (*P* < 0.05). Finally, at 10, 30 and 100 μg/mL, F3 reduced (60%, 55% and 55%, respectively) CXCL2/MIP-2 levels compared to the control (*P* < 0.05; Fig 1D). The difference between CXCL2/MIP-2 levels in Fig 1B and 1D is due to biological variability, and the axis Y was normalized to 2400 pg/mL.

The screening of all fractions through MTT viability and NF-κB activation assays is shown in supporting information S2 Fig (Cytotoxicity and NF-κB activation of the polyphenol-fraction from *Es*e in RAW 264.7 macrophage cells).

### *In vivo* anti-inflammatory assays

**Fig 2:**
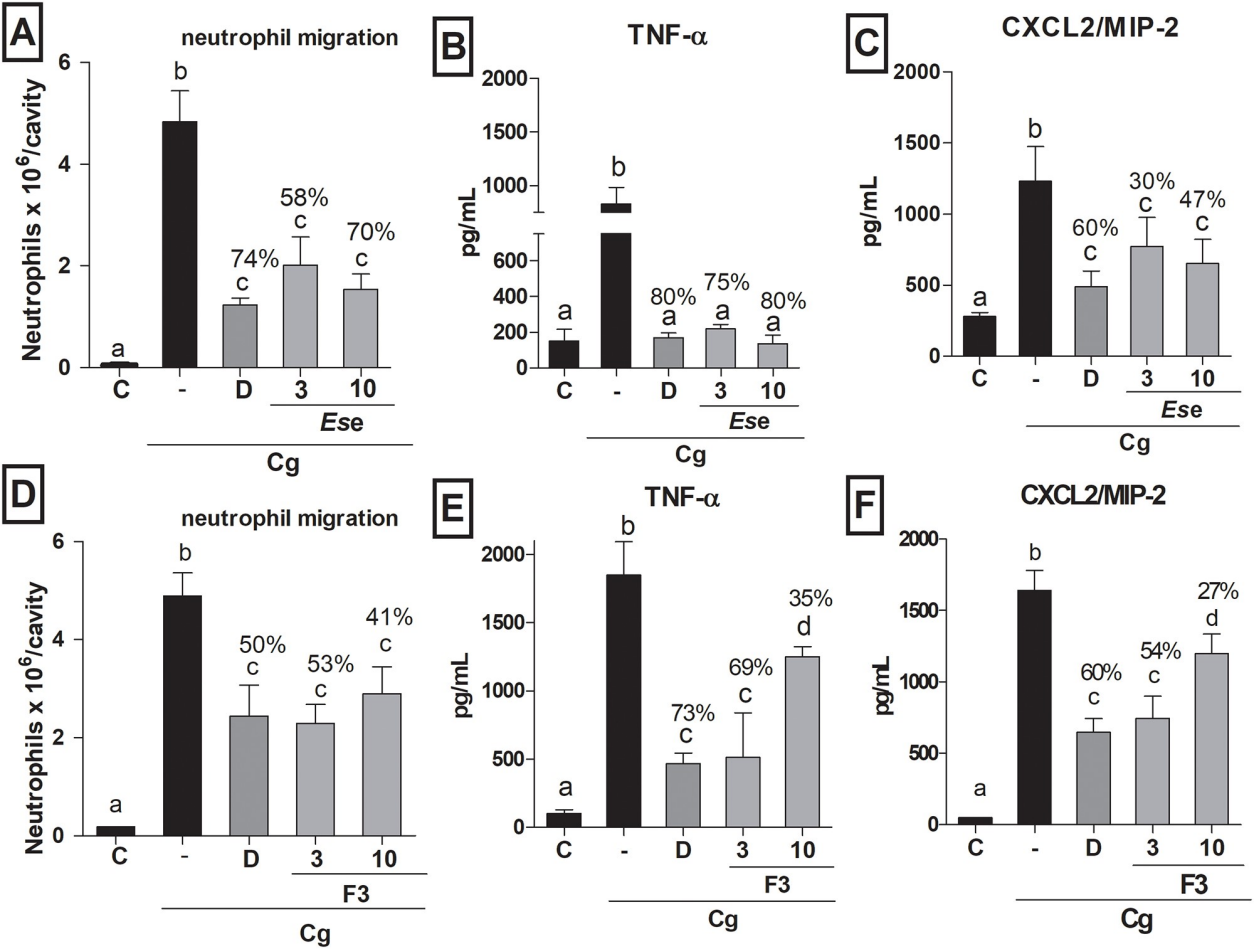
Effects of *Es*e and F3 on neutrophil migration and cytokine release *in vivo*. (A and D) Effects of vehicle (C), carrageenan (-), dexamethasone (D; 2 mg/kg), *Es*e or F3 (3 and 10 mg/kg) on neutrophil migration into the peritoneal cavity of mice induced by i.p. administration of carrageenan (500 μg/cavity, -). (B and E) Effects of the treatments on the release of TNF-α (1.5 h) in mice. (C and F) Effects of the treatments on the release of CXCL2/MIP-2 (3 h) in mice. The results were expressed as mean ± SD, *n* = 5–6. Different letters indicate statistical difference and all groups were compared to each other. One-way ANOVA followed by Tukey’s post-test, *P* < 0.05.

The inhibitory effects of *Es*e and F3 on neutrophil influx were determined *in vivo*. Mice pre-treated with *Es*e at 3 and 10 mg/kg showed a significant decrease in neutrophil influx (58% and 70%, respectively) as compared to those treated with the control carrageenan (Fig 2A). As expected, mice that received dexamethasone showed a significant decrease in neutrophil migration (74%). Interestingly, there was no statistical difference in terms of neutrophil influx between mice treated with *Es*e (at both doses) and those treated with the positive control dexamethasone, a gold-standard corticosteroid widely used in medicine and dentistry (*P* > 0.05). Treatment with *Es*e at 3 and 10 mg/kg also reduced the release of TNF-α (75% and 80%, respectively) and CXCL2/MIP-2 (30% and 47%, respectively) into the peritoneal cavity of mice as compared to each control group, Fig 2B and 2C, respectively.

Mice treated with F3 at 3 and 10 mg/kg showed reduced neutrophil migration (53% and 41%, respectively) in relation to the control group (*P* < 0.05; Fig 2D). In our study, the positive control dexamethasone reduced neutrophil migration by 50% (*P* < 0.05). Mice treated with F3 (3 and 10 mg/kg) showed reduced levels of TNF-α (69% and 35%, respectively) and CXCL2/MIP-2 (54% and 27%, respectively) when compared to the control group (*P* < 0.05; Fig 2E and 2F, respectively). Given the anti-inflammatory potential of *Es*e and F3, both samples were further tested for their antioxidant activity (ROS / RNS scavenging capacity).

### Reactive oxygen/nitrogen species

*Es*e and F3 were tested for their antioxidant activity against peroxyl radical (ROO^•^), superoxide anion (O_2_^•-^), hypochlorous acid (HOCl) and nitric oxide (NO^•^). We investigated the antioxidant activity of *Es*e and F3 against reactive oxygen/nitrogen species, which are biologically equivalent to the human metabolism. As seen in Table 2, while *Es*e was the most active sample to deactivate superoxide anion O_2_^•-^ (172 μg/mL), F3 showed the highest capacity to quench peroxyl radical (680 μmol TE/g extract), hypochlorous acid HOCl (1.13 μg/mL) and nitric oxide NO• (5.05 μg/mL) as compared to *Es*e. The results for the radicals O_2_^•-^, HOCl and NO^•^ are expressed as IC50—which means the amount of substance required to deactivate 50% of the radicals. Overall, F3 seems to have a greater concentration of compounds with both antioxidant and anti-inflammatory properties.

**Table 2:** Antioxidant activity of *Es*e and F3 against peroxyl radical (ROO•), superoxide anion (O_2_•^-^), hypochlorous acid (HOCl) and nitric oxide (NO•).

Sample	ROO• μmol TE/g extract	O_2_^•-^ μg/mL	HOCl μg/mL	NO^•^μg/mL
*Es*e	250 ± 0.008^a^	172.00 ± 14.90^a^	16.68 ± 1.17^a^	11.48 ± 1.84^a^
F3	680 ± 0.02^b^	849.00 ± 4.04^b^	1.13 ± 0.15^b^	5.05 ± 1.07^b^

ROO• is expressed as μm TE/mg of extract (TE = Trolox equivalent), O_2_^•-^, HOCl and NO^•^ are expressed as IC_50_ (μg/mL). The results were expressed as mean ± SD (standard deviation), *n* = 3. Different letters in the same column indicate statistical difference (*P* < 0.05), according to Student’s *t* test.

### Toxicity of *Es*e and F3 in *G*. *mellonella* model

**Fig 3:**
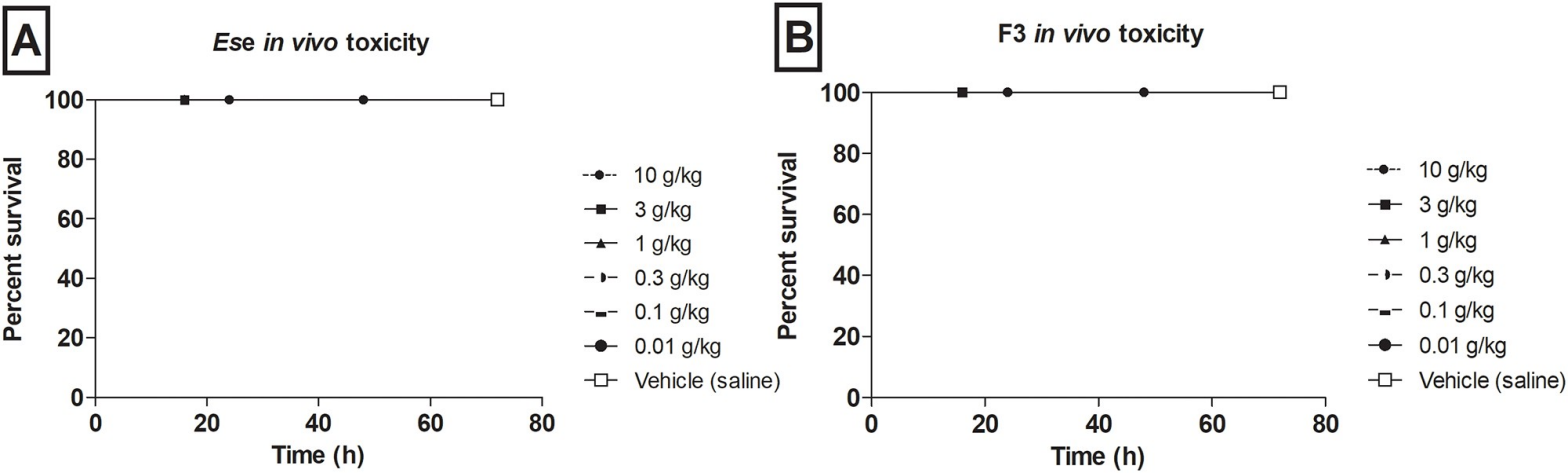
Systemic toxicity of *Es*e and F3 in *G*. *mellonella* larvae model. Larvae were treated with *Es*e and F3 at 0.01, 0.1, 0.3, 1, 3 and 10 g/kg or vehicle (saline) and had their survival monitored up to 72 h (*P* > 0.05, Log-rank test).

*G*. *mellonella* larvae were injected increasing doses of *Es*e and F3 and had their survival monitored for 72 h. As seen in Fig 3, larvae treated systemically with *Es*e and F3 did not demonstrate toxic effects at any tested dose (*P* > 0.05), indicating negligible toxicity of the samples in this *in vivo* model.

## Discussion

It is estimated that approximately 1.7 million deaths per year are related to the low intake of fruits and vegetables, despite the wide cultivation of fruits worldwide. A minimum daily intake of 400 g is required for prevention of chronic diseases and several micronutrient deficiencies [14]. Brazilian native fruit species can be an important ally to one’s health as functional foods while fostering agribusiness and protecting deforestation in threatened rainforests and biomes.

In this bioguided study, we demonstrated that *E*. *selloi* extract and its polyphenol-rich fraction (F3) have (i) anti-inflammatory and (ii) antioxidant activity and (iii) low toxicity *in vivo*. *Es*e and F3 samples were submitted to chemical analysis for tentative identification of compounds based on exact masses, MS/MS spectra and molecular formulas. Gallic acid (*m/z* 169.0105), identified in F3, is an ion produced at *m/z* 125 due to loss of a CO_2_ [15]. Another compound presented a pseudomolecular ion [M-H]^−^ at *m/z* 359.1259, yielding MS2 fragments at *m/z* 197 (loss of a hexosyl moiety; [syringic acid-H]^−^) and 153 (base peak; [syringic acid-H-CO_2_]^−^), suggesting that it could be a syringic acid hexoside in *Es*e and F3 [16]. The fragments corresponding to *m/z* 385.1409 were tentatively identified as isomers of sinapic acid-*O*-hexoside [17].

(Epi)catechin (*m/z* 289.0726) (flavanol class) and (epi)catechin derivative (*m/z* 401.0886) were also found in *Ese*, but not in F3. These compounds are stereoisomers characterized by the molecular ion [M-H- 289]-. The fragments of MS2 corresponding to *m/z* 245, *m/z* 205 and *m/z* 179 indicate that the compound is (epi)catechin [12,18]. The ion *m/z* 245 may result from a loss of a CO_2_ group [M-H-44]- by decarboxylation or by CH_2_CHOH- group loss [15].

Ellagic acid (*m/z* 300.9990), which was detected in *Es*e, belongs to the class of organic compounds known as ellagitannin. This compound is well known for its antioxidant activity with free radical scavenging properties [19]. Ellagitannin compounds were found in both samples, although galloyl-HHDP-hexoside (*m/z* 633.0559) was detected only in F3. Two di-HHDP-galloyl-glucose (*m/z* 935) isomers and three di-HHDP-galloyl-glucose derivate (*m/z* 467 [M-2H]-2) isomers were identified in *Es*e and F3, which presented the fragments *m/z* 633 (M-HHHDP) and *m/z* 301 (ellagic acid) [18,20,21]. Apigenin-7-*O*-glucoside (*m/z* 431.1810) was detected only in F3 based on the *m/z* 269 fragment, which is typical of apigenin [22].

The following flavonols were identified in *Es*e and F3: quercetin-*O*-hexoside (*m/z* 463); quercetin-*O*-(*O*-galloyl)-hexoside (*m/z* 615.0996), which produced *m/z* 179 and 151 corresponding a quercetin fragmentation pattern; quercetin-3-malonylglucoside (*m/z* 549.0889), which presented the *m/z* 301 fragment, a typical ion following the loss of a hexose molecule [M-H-162]- [4,23,24]. Finally, the kaempferol-3-*O*-glucoside (*m/z* 447.0940) was identified in *Es*e and F3 composition [25].

The compound quercetin-*O*-acethylhexoside, present only in *Es*e, was tentatively identified as quercetin-acylated-hexoside based on literature data. This compound showed *m/z* 505.0994 with fragmentation at *m/z* 463 due to loss of an acetyl group (42 Da) and *m/z* at 301 due to loss of a hexoside residue (162 Da) [26].

It is worth noting that some compounds were present in F3, but not in *Es*e. This can be explained by the fact that F3 was obtained through a fractionation process, that is, chemical purification, in a way that compounds previously undetectable become more concentrated and therefore detectable by LC-ESI-QTOF-MS.

Once *Es*e and F3 were chemically characterized, we next tested both samples for their anti-inflammatory activity in different models. Inflammation is an orchestrated host response involving different cell types, such as macrophages. The inflammatory response leads to an intracellular increase of ROS and RNS by macrophages, which can activate the NF-κB intracellular pathway [27,28]. Once activated, this nuclear factor initiates a series of cascade reactions resulting in translocation of p65 and p50 dimers to the macrophage nucleus and ultimately induce gene transcription and release of inflammatory mediators, such as TNF-α and CXCL2/MIP-2 [27,28]. Subsequently, inflammatory mediators increase protein expression on the surface of endothelial cells (selectins and integrins) that control the neutrophil flow into the inflammatory focus [4].

In our study, *Es*e and F3 reduced NF-κB activation in macrophages, decreased the release of TNF-α and CXCL2/MIP-2 and, consequently, mitigated neutrophil migration in mice. Some chemical compounds as ellagitannin identified in *Es*e and F3, have been shown to inhibit NF-κB activation and to modulate neutrophil influx during inflammation [13,21,29]. Quercetin and kaempferol are known to reduce NF-κB activation, TNF-α and IL-1β release, as well as to decrease leukocyte recruitment and oxidative stress [5,13,30–32].

Our findings show that *Es*e and F3 modulated neutrophil migration into the inflammatory focus. Although *Es*e is a chemically crude extract, a complex mixture of bioactive compounds at low concentration, its inhibitory activity on neutrophil influx did not differ from that of the gold standard dexamethasone, which is a pure monodrug. These findings illustrate the potential of *E*. *selloi* as a functional food to reduce or prevent inflammatory diseases and promote health.

In two similar studies, the extracts of *E*. *leitonii* seeds and *E*. *brasiliensis* pulp (also from the *Eugenia* genus) reduced neutrophil influx into the inflammatory focus, with no difference from dexamethasone, and also decreased NF-κB activation [533]. Interestingly, these three species (*E*. *leitonii*, *E*. *brasiliensis* and *E*. *selloi)* share two compounds in their chemical composition, ellagic acid and quercetin, which may be responsible for the anti-inflammatory activity observed [33,34].

Despite the benefits of the inflammatory response, an exacerbated presence of neutrophils and other cells generating excessive ROS / RNS production may create an imbalance in pro- and antioxidants, ultimately damaging tissues, DNA, proteins, and other host components [35].

Here, *Es*e and F3 were further tested for their ROS / RNS scavenging activity. Our findings indicate that F3 was more effective than *Es*e in this regard, despite the fact that the fractionation process was guided by the anti-inflammatory data. *Es*e was more effective in quenching the superoxide anion (O_2_•) compared to F3, which can be explained by the synergism among the phenolic compounds present in the crude extract. Four *Eugenia* spp. were previously tested for their O_2_• radical scavenging activity and the authors reported IC_50_ ranging from 215 to 402 μg/mL, indicating that *E*. *selloi* is more potent than those native species. [5]. The superoxide anion contributes to metabolic oxidative stress, resulting in cell damage and genomic instability. Hence, the inhibition of this radical can be an important strategy to prevent diseases related to oxidative stress [35].

The ORAC (oxygen radical absorbance capacity) assay is a direct method to measure hydrophilic and lipophilic chain-breaking antioxidant capacity against the peroxyl radical, generated by AAPH via hydrogen atom transfer reactions [35]. In the context of inflammation, ROS present in the lipid tissue are converted into peroxyl radical and may cause cell membrane damage, neoplasia and, most likely, several inflammatory chronic and degenerative diseases [35,36]. Our findings showed that the peroxyl radical scavenging capacity of F3 was three-fold greater than that of *Es*e. A previous study testing gallic acid showed ORAC values of 7.88 mol TE/g, which is 32-fold and 12-fold more potent than that of *Es*e and F3, respectively [37]. Here, isomers of quercetin were identified in both samples and were previously shown to have antioxidant activity against peroxyl radical [38]. The potent peroxyl radical scavenging capacity of *Es*e and F3 might be due to the chemical compounds present in their composition, such as hydroxybenzoic acid and flavonols.

We next determined the ROS (hypochlorous acid—HOCl) scavenging activity of the samples. *Es*e and F3 exhibited different IC_50_ values, which indicated that F3 was approximately 16-fold more potent than *Es*e to scavenge HOCl. Although *Es*e is a crude extract, its HOCl radical scavenging activity was comparable to that of Trolox (IC_50_ of 134 μg/mL), a standard drug [39]. As compared to other native fruits, *Es*e and F3 were more active than *E*. *brasiliensis* and *E*. *leitonii* pulp (IC_50_ of 42 and 109 μg/mL, respectively) [5].

The samples were further tested for their nitric oxide (NO•) scavenging capacity. In this regard, F3 was approximately 2-fold more effective than *Es*e. A previous study investigated the NO• scavenging capacity of *E*. *stipitata*, a Brazilian native fruit belonging to *Eugenia* genus, and observed an equivalent activity (IC_50_ of 6.95 μg/mL) [4]. We detected the presence of quercetin derivatives in both samples and this flavonoid is known to be more potent than other antioxidant nutrients, such as vitamin C and vitamin E [40]. In another study, the authors evaluated the NO• scavenging capacity of *Monodora myristica* extract and observed activity (IC_50_ of 171.38 μg/mL) and this antioxidant activity could be related with the presence of syringic acid, also identified in *Es*e and F3 [41].

The different potency observed in the samples can be due to the synergism and chemical composition present in the crude extract and its fraction.

This is the first report on the ROS / RNS scavenging activity *E*. *selloi* extract and its purified fraction (F3). ROS and RNS are important components in the inflammation process for their capacity to promote cell / tissue damage in the organism. The question that remains is how and how much ROS and RNS contribute to the inflammatory response. Thus, controlling the balance between oxidation and anti-oxidation through exogenous antioxidants obtained from the diet (*e*.*g*., daily fruit intake) is a promising strategy to prevent the onset of inflammatory diseases [10,40].

This is also the first report on the *in vivo* systemic toxicity of *Es*e and F3 in *G*. *mellonella* larvae. This is a simple, a low-cost, and validated model widely used, because the results obtained in this model correlate with those observed in mammals [42]. In our study, we did not find the lethal dose (LD_50_) of the samples even when testing a dose 1000 times higher (10 g/kg) than that used in mice (10 mg/kg). In a previous study with other Brazilian native fruit *(E*. *leitonii* seed and *E*. *brasiliensis* leaf extracts), the authors showed LD_50_ of 1.5 g/kg for both extracts using *G*. *mellonella* model. However, we evaluated the pulp extract in our study, which seems to have negligible toxicity in this model as compared to other parts of *Eugenia* spp. fruits [13].

## Conclusions

*E*. *selloi* has proven to be a good source of bioactive compounds, even better than the best fruits traditionally consumed, and it was further shown to modulate the inflammatory process. This study adds value to the promising Brazilian native fruit that have emerged as a source of bioactive compounds providing benefits for human health (functional foods), contributing to the development of agribusiness for many families, and also contributing to preservation of biodiversity, particularly in the Brazilian Atlantic Rainforest.

## Supporting information

S1 Fig Visual aspect of *Es*e, chemical fractionation, yield and thin layer chromatography. (TIFF)

S2 Fig Cytotoxicity and NF-κB activation of the polyphenol-rich fraction from *Es*e in RAW 264.7 macrophage cells. (TIFF)
