# Supplementary figures and images for: Anti-inflammatory and antioxidant potential, in vivo toxicity, and polyphenolic composition of Eugenia selloi B.D.Jacks. (pitangatuba), a Brazilian native fruit
Source: PLoS One. 2020 Jun 9;15(6):e0234157. doi: 10.1371/journal.pone.0234157 (PMC7282636; doi:10.1371/journal.pone.0234157)

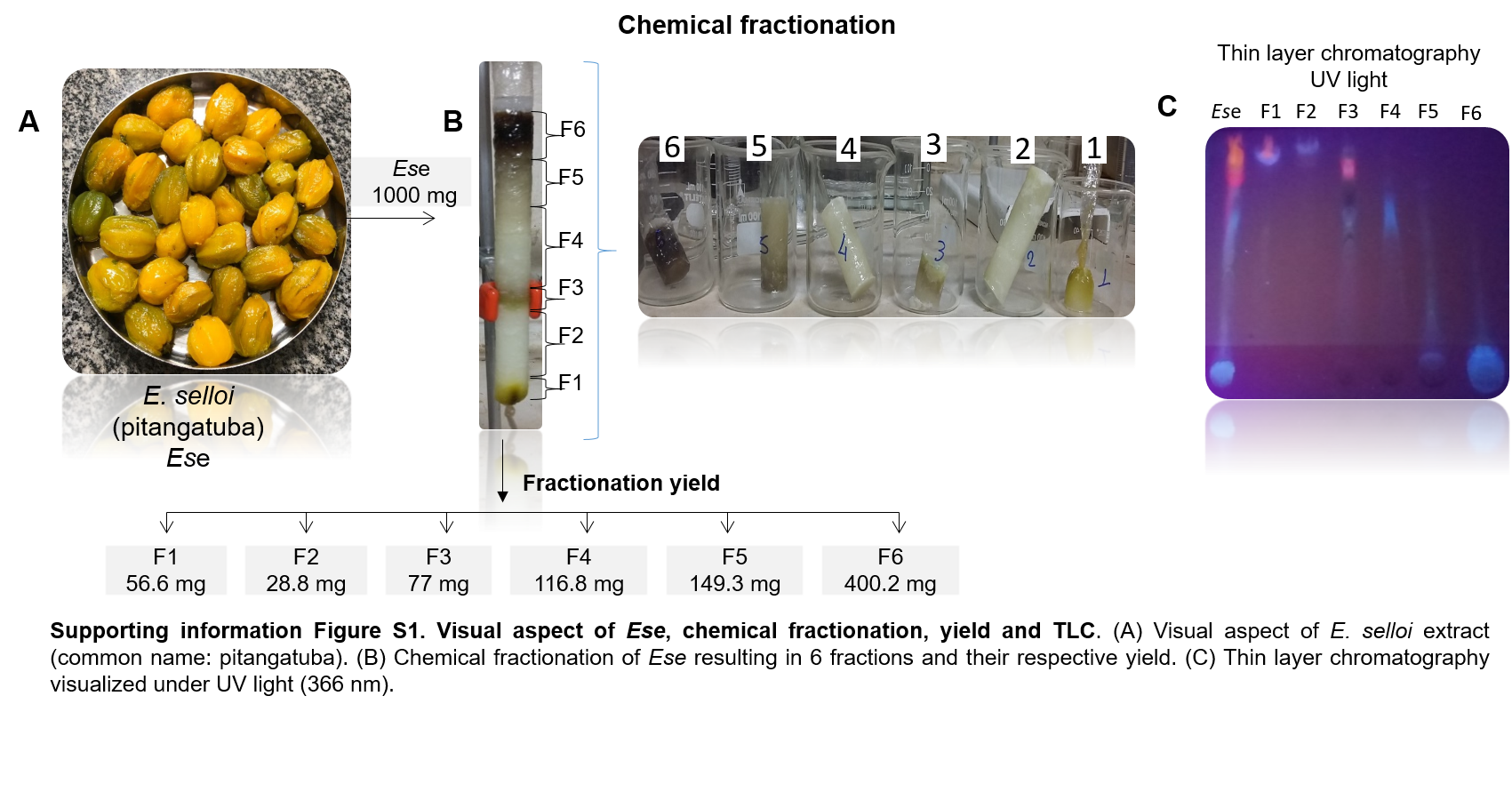

Supplement: S1 Fig — (TIFF) [file pone.0234157.s001.tiff]

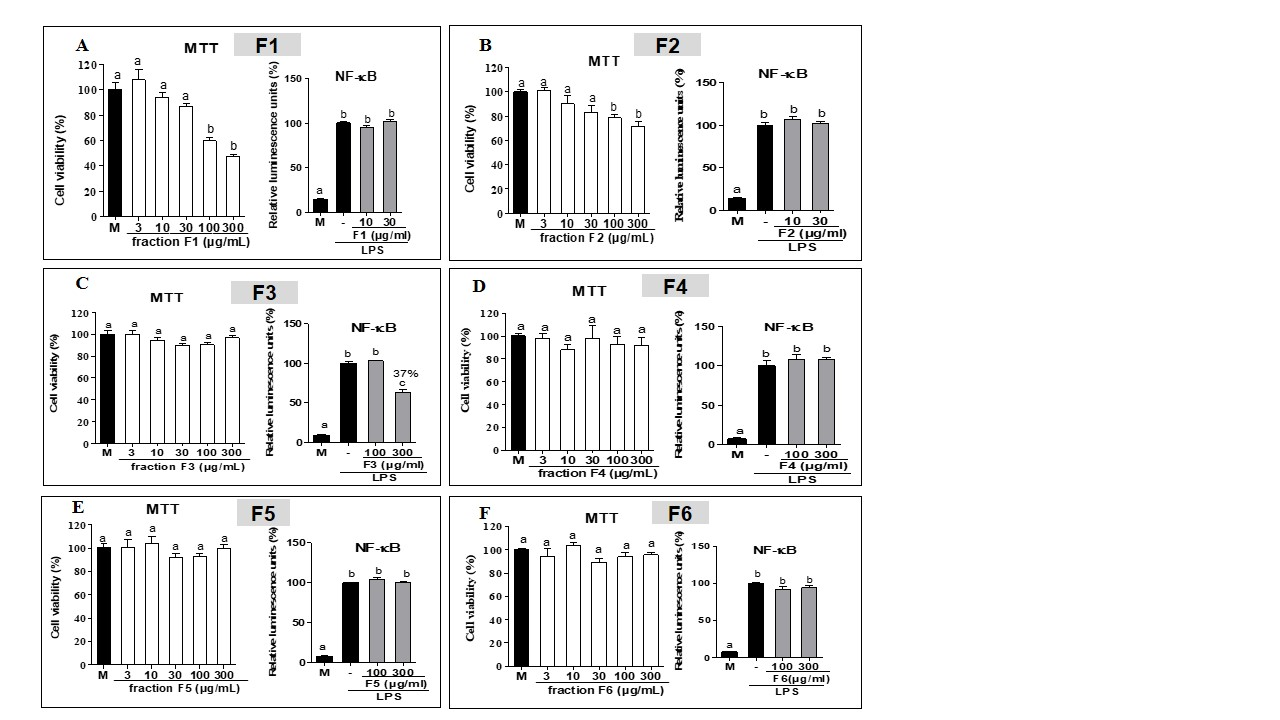

Supplement: S2 Fig — (TIFF) [file pone.0234157.s002.tiff]
